# Research on recycling value grading and real-time perception of rock debris from TBM tunneling

**DOI:** 10.1038/s41598-025-95072-0

**Published:** 2025-04-03

**Authors:** Weiqi Yue, Weilin Su, Zhanfei Gu, Xiao Qu

**Affiliations:** 1 School of Civil Engineering and Environment, Zhengzhou University of Aeronautics, No. 15 Wenyuan West Road, Zhengzhou, 450046 Henan China; 2 Yellow River Engineering Consulting Co., Ltd, No. 109 Jinshui Road, Zhengzhou, 450003 Henan China

**Keywords:** TBM tunneling, Rock debris, Recycling value grading, Real-time perception, Machine learning, Civil engineering, Environmental impact

## Abstract

During the construction of TBM tunnels, a substantial quantity of rock debris is generated, leading to significant land occupation and environmental pollution. Recycling rock debris into construction materials and other resources emerges as a viable solution to these problems. To realize the continuous classified storage and disposal of tunnel rock debris, this research explores the four-level processing network, establishes an objective function for evaluating the recycling value of tunnel rock debris during TBM tunneling, and grades the recycling value by calculating the weight and similarity of their performance indicators (uniaxial compressive strength, content of acicular and flattened particles, mud content, and crushing index) through the TOPSIS method. Through correlation and weight analysis, we identify five key characteristics, i.e. cutterhead torque, tool penetration, cutterhead thrust, advancing rate, and support shoe pump pressure, to conduct real-time perception of the recycling value level of rock debris. Leveraging a comprehensive database that encompasses both tunnel rock debris performance indicators and TBM tunneling parameters, perception models are constructed using different machine learning algorithms. After Bayesian hyperparameter optimization, the perception models based on CART, SVM, KNN, and ANN demonstrate accuracies of 67.5%, 80.0%, 82.5%, and 83.8% respectively. Notably, the hyperparameter optimization significantly enhances the accuracy of the ANN perception model. When applying the optimized ANN-based rock debris recycling value grade perception model to TBM tunnel engineering, the tested perception accuracy rate stands at 83.3%, demonstrating its effectiveness and potential for practical applications. This approach provides valuable guidance for the graded storage and efficient recycling of tunnel rock debris and helps to alleviate the pollution problem.

## Introduction

The rock tunnel boring machine (TBM) is widely used in various engineering projects such as water tunnels^1–5^, railway tunnels^6,7^, and highway tunnels^8^ due to its advantages of high efficiency, safety, and excellent construction environment. Especially for long and large tunnels, the choice of the TBM method for construction offers unparalleled advantages in terms of construction costs and schedule compared to the traditional drilling and blasting method^9–11^. However, a large amount of rock debris is generated during the construction of TBM tunnels. If not properly handled, it will occupy a significant amount of land resources and release harmful substances such as heavy metals and radioactive materials into the soil, water sources, and even the air, causing environmental pollution^12^. Recycling, which transforms rock debris into construction materials and other resources, is an effective means to address these issues while achieving significant economic and ecological benefits^13,14^.

The most common practice for rock debris generated during tunnel construction is to mix and stack together rock debris with different components and performances. These rock debris, which varies greatly in their recycling value, are difficult to utilize directly. Eventually, they have to be discarded as waste. Once the rock debris is produced from the tunnel construction site, its strength, composition, and other performance indicators should be tested immediately to match the recycling direction. Then, based on the test results, the rock debris should be classified and utilized as resources. However, conventional rock debris testing methods are difficult to sample, involve multiple indicators, and take a long time, which cannot meet the requirements of rapid transportation of rock debris and rapid construction of the TBM tunnel^15^. Therefore, rapid classification based on the composition and performance of TBM tunnel rock debris is the key to its recycling.

Machine learning, a crucial component of artificial intelligence, finds extensive application in fields like computer vision, natural language processing, and data classification and regression^16–20^. In the TBM tunnel construction, machine learning techniques can leverage engineering data such as tunneling parameters and stratigraphic information to comprehensively consider the diverse and complex factors encountered during the excavation process. This approach enables intelligent perception of the TBM construction environment, while also providing the capability for real-time dynamic updates and predictions^21–24^.

To categorize the recycling value levels and utilization directions of the TBM tunnel rock debris, this research establishes an objective function for evaluating the recycling value, providing a feasible way to link the physical and mechanical properties of rock debris with its recycling value. Drawing upon the performance indicators and compositional information, the recycling value of rock debris is graded by calculating the weight and similarity of their performance indicators through the TOPSIS method. To assess the recycling value grade of the debris during TBM tunneling, perception models of rock debris recycling value are constructed based on machine learning algorithms and the TBM boring parameter database. This approach provides valuable guidance for the graded storage and efficient recycling of tunnel rock debris.

## The recycling direction and value of the TBM tunnel rock debris

### Processing network of tunnel rock debris

**Fig. 1:**
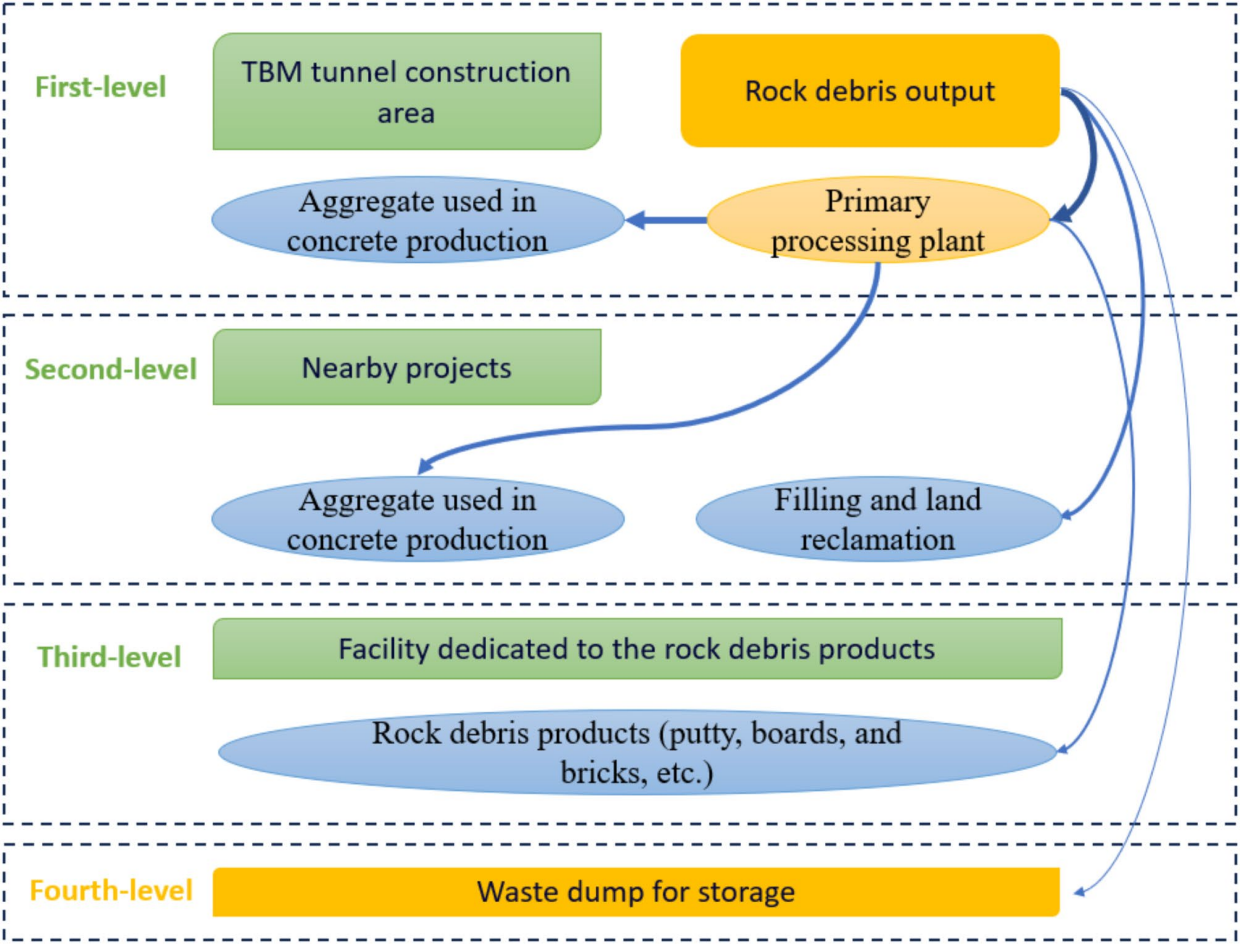
Processing network of tunnel rock debris (the thickness of the arrow indicates the processing priority).

The allocation of tunnel rock debris should adhere to the principle of “sustaining tunnel construction with tunnel resources”, considering the impact of time factors and other demand directions. The comprehensive evaluation indicators should be based on satisfying performance requirements and maximizing the recycling value of rock debris. As shown in Fig. 1, the processing network for tunnel rock debris can be divided into four levels:

The first-level rock debris processing network is located at the top of the entire rock debris processing network, consisting of the tunnel construction area and the primary processing plant for rock debris. The tunnel construction area serves as the output for rock debris, while the primary processing plant serves as the input end for debris, with the function of promptly processing the debris produced in the construction area into coarse aggregate used in concrete production. The primary processing plant should be established near the entrance of the tunnel construction area to achieve “zero transportation distance” and enable the rapid supply of concrete required for the tunnel project.

After supplying the rock debris that meets the requirements in terms of performance and quantity to the first-level processing network, the remaining rock debris will be transported to the second-level processing network. Within the second-level network, there are two primary flow paths for the rock debris. Firstly, the rock debris generated in the tunnel construction area is directly utilized for filling and land reclamation in nearby projects. Secondly, after initial processing at the primary processing plant, the rock debris products are allocated to fulfill the material requirements for aggregate and prefabricated products (e.g., prefabricated beams and columns) in projects unrelated to the current tunnel, thus ensuring efficient utilization of resources across multiple construction sites.

After meeting the demands of both the first-level and second-level processing networks for rock debris, the remaining debris is allocated to the third-level processing network. This level comprises the tunnel construction area and a facility dedicated to the rock debris products (the rock debris deep processing plant, ). Here, the transported rock debris undergoes further processing to transform it into various marketable products such as putty, boards, and bricks.

When the performance of the rock debris does not meet the requirements of the aforementioned three-level processing networks, it needs to be transported to a waste dump for storage, forming the fourth-level processing network for tunnel rock debris management.

### Tunnel rock debris recycling value objective function

The recycling value of tunnel debris is defined as the economic value created per unit volume of debris for tunnel engineering (realized through sales revenue or cost savings within the project). This value needs to be evaluated from the following three perspectives:

1. Processing Cost of Tunnel Debris for Recycling (*C*): This refers to the cost of converting the unit volume of TBM tunnel debris into sand, gravel, market-ready raw materials, fillers, and other products. It is determined by factors such as debris quality, processing techniques, and product requirements.
2. Productivity Rate of Tunnel Debris Products (*r*): This represents the volume of debris products obtained after processing unit volume of tunnel debris through methods such as crushing, washing, and filtering. It is primarily determined by the composition and physical properties of the debris.
3. Unit Price of Tunnel Debris Products (*P*): This is the selling price per unit volume of debris products or the purchase price that replaces the cost of acquiring sand and gravel for the project. It is influenced by debris quality, intended use, and market conditions.
4. Transportation Cost of Tunnel Debris (*T*): This encompasses the expenses associated with transporting unit volume of TBM tunnel debris to waste storage sites, processing plants, and landfilling locations. It is determined by factors such as transportation distance, mode of transportation, and transportation convenience.

Therefore, the recycling value of tunnel debris can be determined by the following function:

1 $${\text{O}}bt=\sum\limits_{{k=1}}^{n} {\left[ {{P_k} - \frac{1}{{{r_k}}}({C_k}+{T_k})} \right]}$$

where *P*_*k*_, *r*_*k*_, *C*_*k*_, and *T*_*k*_ represent the unit price, productivity rate, processing cost, and transportation cost of debris in the *k-*th level of the recycling processing network, respectively. The variable *n* represents the number of tiers in the recycling processing network, and in this context, *n* = 4.

### Tunnel rock debris performance indicators

Based on the requirements for mechanically processed sand and gravel materials used in engineering as stipulated in the “Standard for Construction Quality Acceptance of Railway Concrete Engineering of China“^25^, four parameters, namely uniaxial compressive strength (*UCS*), content of acicular and flattened particles (*AFC*), mud content (*MC*), and crushing index (*CI*), are selected in this paper as the performance evaluation indicators for tunnel rock debris. The testing methods, operational steps, and required instruments and equipment for each indicator are listed in Table 1.

**Table 1:** Rock debris performance indicators and test methods.

Parameters	Test methods	Steps	Instruments
*UCS*	Point load compression test	1. Select representative test points on the specimen, ensuring that the test points avoid defects such as joints and cracks 2. Place the point load meter on the test points, ensuring that the meter is in close contact with the surface of the specimen 3. Apply the load and record the load value at the time of specimen failure 4. Calculate the point compressive strength of the rock material based on the point load test formula	Point load testing machine
*AFC*	Gauge measurement method	1. Weigh a specified amount of sample to the nearest 1 g, and then sieve it according to the specified particle size 2. Identify the aggregates that are visually determined as potentially acicular or flattened particles. If the length of a particle is greater than the corresponding distance on the Acicular Particle Gauge and it cannot pass through, it is considered an acicular particle 3. For non-acicular particles, the ones that can pass through the corresponding holes on the Flattened Particle Gauge are considered flattened particles 4. Weigh the total mass of the picked-out acicular particles and flattened particles	Acicular and flattened particle gauge
*MC*	Cleaning and sieving method	Dry the sample to constant weight in air at a temperature of 110–115 °C 2. Weigh a certain amount of the sample and pour it into a washing container. Add clean water and stir thoroughly. After soaking for some time, wash the sample in the water to separate dust, silt, clay, and other impurities from the aggregate particles 3. Pour the muddy water onto a sieve to filter out particles smaller than the specified particle size. Then, dry and weigh the mass of the residue left on the sieve	Drying oven, Balance, and Standard sieve
*CI*	Crushing and sieving method	1. Dry the sample to eliminate the influence of moisture on the test results 2. Fill the aggregate particles into a standard cylindrical mold, ensuring that the sample is evenly distributed in the mold 3. Apply the specified pressure (400 kN) to the mold and maintain it for 5 min 4. Unload the pressure, remove the crushed sample from the mold, sieve out the fine particles crushed by using a sieve with a pore size of 2.36 mm, and weigh the sample mass remaining on the sieve	Universal testing machine, Cylindrical mold, Balance, and standard sieve

## Recycling value grades for TBM tunnel rock debris

Drawing upon a practical TBM tunnel project, we conducted tests on the point load compressive strength, acicular or flattened particle content, mud content, and crushing index of the rock debris generated during TBM tunneling in various tunnel sections. Concurrently, the TBM tunneling parameters were monitored and recorded automatically in the tunnel sections where the rock debris was produced. After data cleaning and standardization, a comprehensive database encompassing both the performance indicators of tunnel rock debris for recycling and the tunneling parameters of TBM was established. This database serves for the categorization and identification of the recycling value grades of TBM tunnel rock debris.

### Project introduction and data acquisition

1. Project introduction

**Fig. 2:**
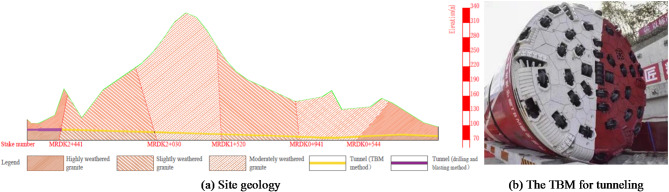
Project site geology and the TBM for tunneling.

The access tunnel for subway trains entering and leaving parking areas of subway Line 6 in Shenzhen, China, is excavated by a double-shield TBM and it mainly passes through granite with different degrees of weathering, as shown in Fig. 2a. TBM advancing distance of 816 m (stake number: MRDK2 + 446 ~ MRDK1 + 630) is selected for this research, the buried depth of which ranges from 41.4 m to 249.7 m.

(2) Data acquisition

A double-shield TBM was utilized in the excavation of the tunnel, as shown in Fig. 2b, and the outer diameter of the cutterhead of the TBM is 6.5 m. During TBM boring operations, a significant amount of equipment parameters were captured through the data acquisition system, encompassing a total of 144 parameters from various systems such as the main engine, drive, hydraulics, reducer, cutterhead, support shoes, and transport belts. Considering the direct influence of the performance of the tunnel rock debris on the TBM, 7 key indicators were selected for establishing the TBM tunneling parameters database: cutterhead torque (*T*), cutterhead power (*Ph*), cutter penetration (*Prev*), cutterhead thrust (*Tr*), advancing rate (*Ar*), thrust oil pressure (*Pc*), and support shoe pump pressure (*Pb*). These indicators exhibit a strong correlation with the performance of tunnel rock debris.

**Fig. 3:**
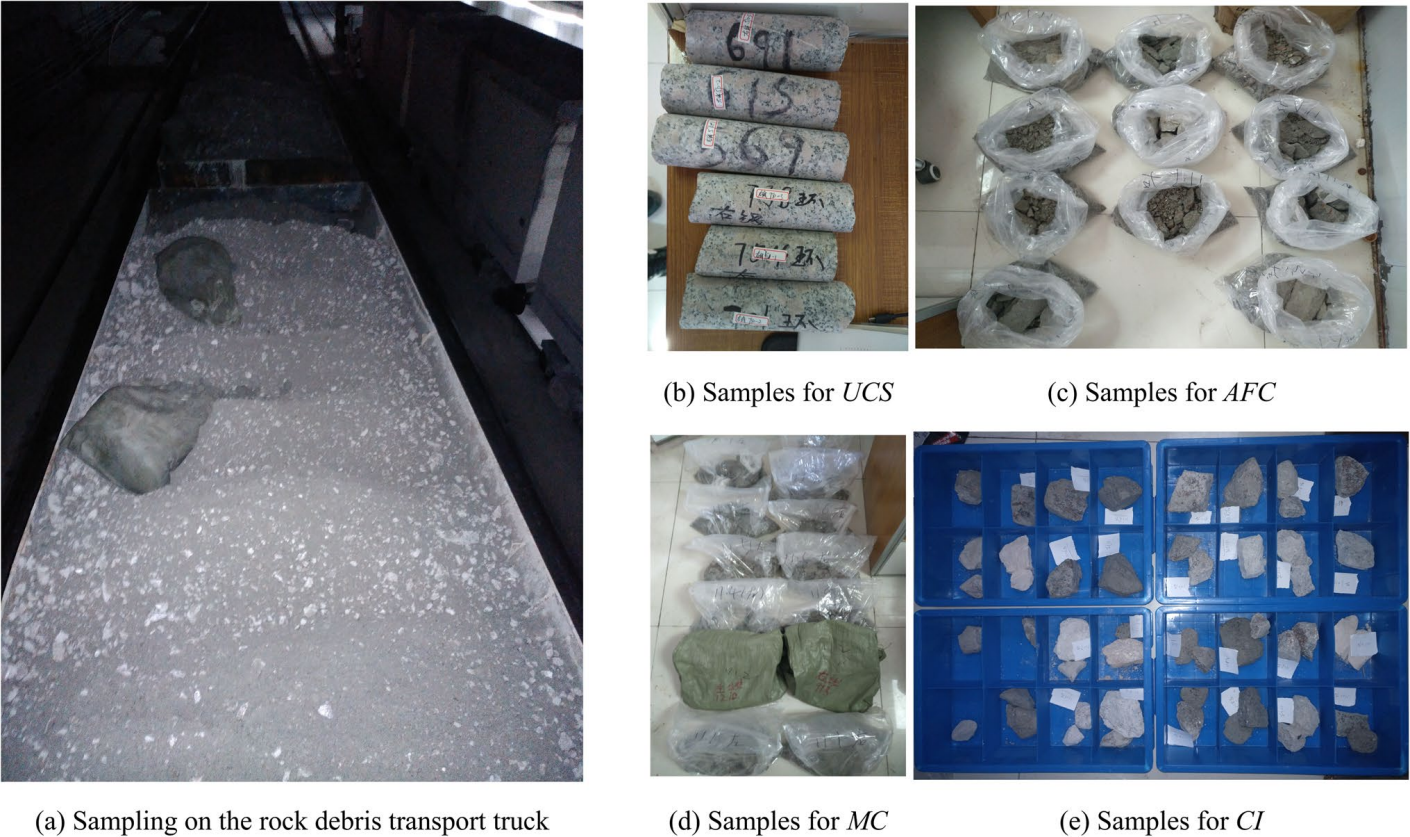
Sampling for rock debris performance indicator tests.

After one cycle of TBM tunneling, the rock debris for the test is directly sampled from the debris transport truck (Fig. 3a) or tunnel wall drilling. To ensure the variability of the performance indicators of the rock debris samples, the sampling interval for rock debris is set at 20 to 50 m along the direction of TBM tunneling. To obtain more accurate performance indicators of the rock debris, 6 groups of samples were taken from each tunnel section for tests (Fig. 3b–e).

By collecting and analyzing the TBM tunneling parameters that correspond to the performance indicator samples related to the recycling value of tunnel rock debris, a comprehensive sample set of TBM tunneling parameters was established. This sample set is detailed in Table 2.

**Table 2:** Performance indicators of rock debris samples and corresponding TBM tunneling parameters.

Categories	Parameters	Number of samples	Maximum	Minimum	Average
Performance indicators	*UCS*/MPa	110	104.0	24.1	54.1
	*AFC*	110	1.81	0	0.69
	*MC*	110	0.150	0.007	0.036
	*CI*/m	110	46.9	3.4	21.3
Tunneling parameters	*T*/kN m	110	617.7	60.1	379.7
	*Ph*/kW	110	587.5	16.8	330.2
	*PRev*/(mm/rev)	110	9.8	0.7	3.7
	*Tr*/kN	110	6465.7	2803.7	5057.1
	*Ar*/(mm/min)	110	48.4	4.7	28.2
	*Pc*/kpa	110	2984.5	612.5	1562.4
	*Pb*/kPa	110	315.6	188.1	267.3

### The TOPSIS for the recycling value of rock debris

The TOPSIS (Technique for Order Preference by Similarity to an Ideal Solution) is a commonly used multi-objective evaluation method that assesses samples by measuring the distance (degree of closeness) between the feature attribute values of the samples and the optimal solution (positive ideal solution) as well as the worst solution (negative ideal solution). In this paper, the positive and negative ideal solutions for the recycling value grades of tunnel rock debris are established, and the evaluation index weight coefficients and proximity degree are calculated to evaluate the recycling value of TBM tunnel rock debris.

1. Normalization of performance indicators of the sample

Assuming that the sample size of TBM tunnel rock debris is *m*, and each sample possesses *n* performance indicators related to recycling value, the initial evaluation matrix for the recycling value of the rock debris can be expressed as:

2 $$X={({x_{ij}})_{m \times n}}$$

where *X* represents the initial evaluation matrix for the recycling value of the rock debris samples; *x*_*ij*_ represents the parameter values of the sample. Since *x*_*ij*_ in the initial evaluation matrix has different dimensions and ranges of values, it is necessary to normalize *x*_*ij*_ and matrix *X* to eliminate their influence:

3 $$r{}_{{ij}}=\frac{{{x_{ij}} - \hbox{min} ({x_{ij}})}}{{\hbox{max} ({x_{ij}}) - \hbox{min} ({x_{ij}})}}$$

4 $$R={({r_{ij}})_{m \times n}}$$

where max(*x*_*ij*_) and min(*x*_*ij*_) represent the maximum and minimum values of the performance indicator values for the samples, respectively; *r*_*ij*_ represents the normalized value of the parameters for the rock debris sample, where *r*_*ij*_ ∈ [0,1]; *R* represents the normalized evaluation matrix for the recycling value of the rock debris samples.

(2) Decision matrix and parameter weights

The performance indicators of tunnel rock debris have varying degrees of impact on its recycling value, meaning that each of the *n* parameters carries a distinct weight in assessing the recycling value of rock debris samples. Given the weight index vector ***W*** = [*w*_1_,*w*_2_,…,*w*_n_], a normalized weighted decision matrix can be formulated to evaluate the recycling value of rock debris from TBM tunnels.

5 $$Z={({z_{ij}})_{m \times n}}=\left[ \begin{gathered} {w_1}{r_{11}}\;\;\;{w_2}{r_{12}}\;\;\; \ldots \;\;\;{w_n}{r_{1n}} \hfill \\ {w_1}{r_{21}}\;\;\;{w_2}{r_{22}}\;\;\; \ldots \;\;\;{w_n}{r_{2n}} \hfill \\ \;\;\; \vdots \;\;\;\;\;\;\;\;\; \vdots \;\;\;\;\;\; \ddots \;\;\;\;\;\; \vdots \hfill \\ {w_1}{r_{m1}}\;\;\;{w_2}{r_{m2}}\;\;\; \ldots \;\;\;{w_n}{r_{mn}} \hfill \\ \end{gathered} \right]$$

where the weighted impact of the *j-*th performance indicators on the recycling value of the *i-*th rock debris sample is represented as *z*_*ij*_.

(3) Proximity degree

The expressions for the positive and negative ideal solutions of the normalized weighted decision matrix for sample recycling value are respectively:

6 $$\left\{ \begin{gathered} {{\varvec{Z}}^+}=[\hbox{max} ({z_{ij}})]=[z_{1}^{+},\;\;z_{2}^{+}, \ldots ,z_{n}^{+}] \hfill \\ {{\varvec{Z}}^ - }=[\hbox{min} \;({z_{ij}})]=[z_{1}^{ - },\;\;z_{2}^{ - },\ldots ,z_{n}^{ - }] \hfill \\ \end{gathered} \right.$$

where ***Z***^+^ and ***Z***^−^ represent the positive and negative ideal solutions, respectively. The expressions for calculating the distances between the parameters of a rock debris sample and the positive and negative ideal solutions are as follows:

7 $$\left\{ \begin{gathered} D_{i}^{+}=\sqrt {\sum\limits_{{j=1}}^{n} {{{({z_{ij}} - z_{{_{j}}}^{+})}^2}} } \hfill \\ D_{i}^{ - }=\sqrt {\sum\limits_{{j=1}}^{n} {{{({z_{ij}} - z_{{_{j}}}^{ - })}^2}} } \hfill \\ \end{gathered} \right.$$

where *D*_*i*_^+^ and *D*_*i*_^−^ represent the positive and negative ideal solutions for the recycling value of the *i-*th rock debris sample, respectively. The expression for the proximity degree of the recycling value of this sample is as follows:

8 $${S_i}=D_{i}^{ - }/(D_{i}^{+}+D_{i}^{ - })$$

where *S*_*i*_ represents the proximity degree of the recycling value of the TBM tunnel rock debris for the *i-*th rock debris sample.

### Application of TOPSIS

1. Value assignment of sample performance indicator weights

According to the descriptions in 2.1 and 2.2, the key to calculating the proximity degree of rock debris recycling value using the TOPSIS method is to determine the value parameters *P*_*k*_, *r*_*k*_, *C*_*k*_, and *T*_*k*_ in the objective function (Eq. 1) as well as the weight vector ***W*** of the decision matrix. Among them, *P*_*k*_, *C*_*k*_, and *T*_*k*_ are influenced by numerous factors such as tunnel construction site layout and treatment processes. Taking the TBM tunnel project introduced in this article as an example, the values of *P*_*k*_, *C*_*k*_, and *T*_*k*_ under different recycling value grades *k* are shown in Table 3. On the other hand, the output rate *r*_*k*_ is primarily determined by the quality of the rock debris, and the values of *r*_*k*_ for different samples may vary (*r*_1_ + *r*_2+_*r*_3+_*r*_4_ = 1). The weight vector ***W*** needs to be determined based on the correlation between the surrounding rock parameters and their recycling value.

**Table 3:** Value assignment parameters *P*_*k*_, *r*_*k*_ and *C*_*k*_ under different recycling value grades.

Parameters	k = 1	k = 2	k = 3	k = 4
*P*_*k*_ (Yuan per ton)	120	96	108	0
*C*_*k*_ (Yuan per ton)	41	41	50	0
*T*_*k*_ (Yuan per ton)	3.5	15	20	12

The study established the correlation between the uniaxial compressive strength (*UCS*), content of acicular and flattened particles (*AFC*), mud content (*MC*), and crushing index (*CI*), and the recycling value objective function, respectively. The correlation of each feature was evaluated using the correlation coefficient *R*^2^, as shown in Fig. 3.

**Fig. 4:**
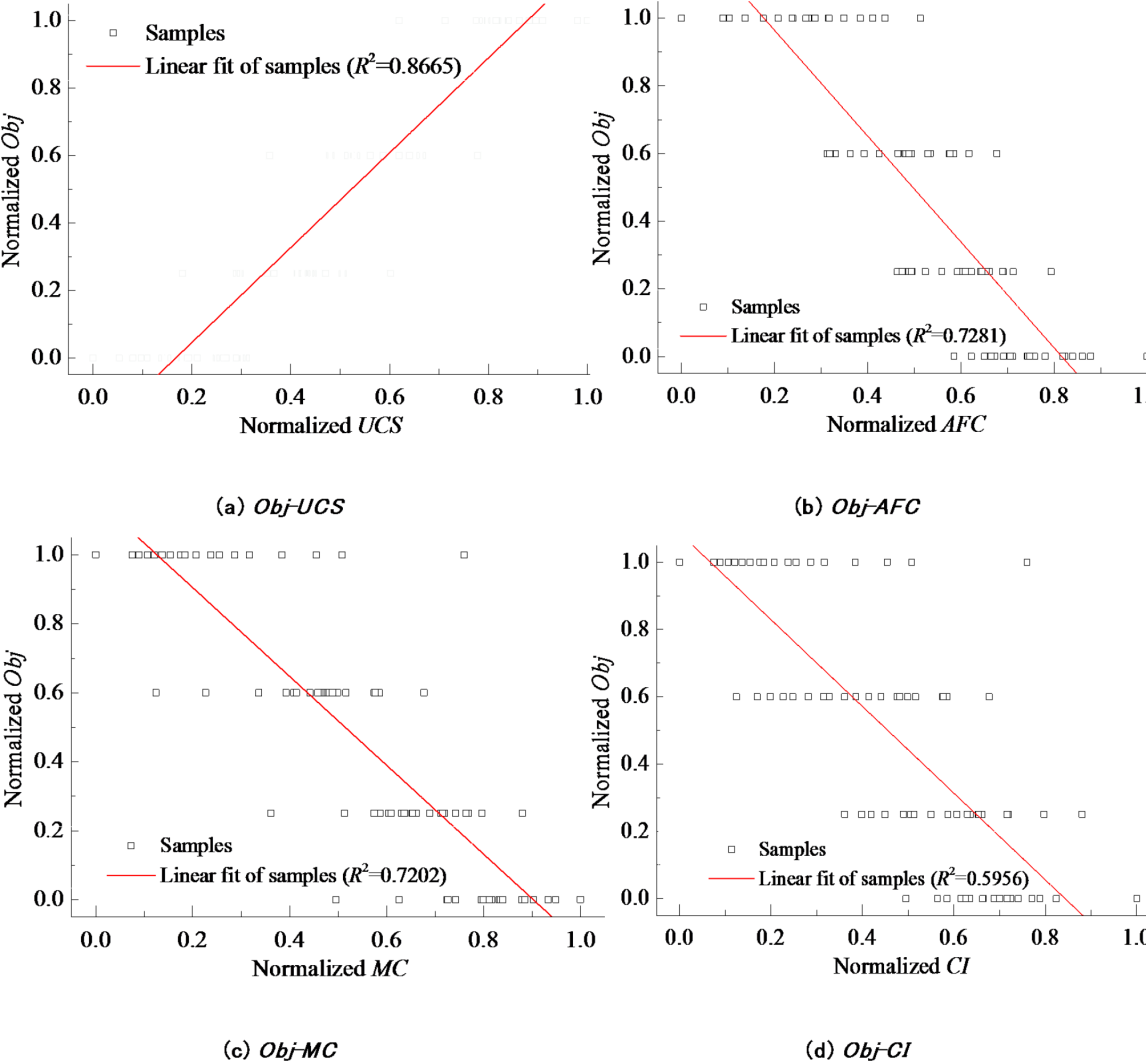
The correlation between the performance indicators and the recycling values.

Based on Fig. 4, the performance indicators of the TBM tunnel rock debris all follow a normal distribution with a relatively large variance, which leads to a roughly linear correlation between the various performance indicators of tunnel rock debris and the normalized objective function for recycling value. By analyzing the correlation coefficient *R*^2^ between each indicator and normalized *Obf* depicted in Fig. 4, we calculate the parameter weights for evaluating the recycling value of rock debris, as presented in Table 4.

**Table 4:** Parameter weights for evaluating the recycling value of rock debris.

Indicators	Performance indicators			
	UCS	AFC	MC	CI
*R* ^2^	0.8665	0.7281	0.7202	0.5956
Parameter weights	0.2977	0.2502	0.2475	0.2046

(2) Establishment of the TOPSIS model

By substituting the weight vector of sample performance indicators ***W*** = [0.2977, 0.2502, 0.2475, 0.2046] into the initial evaluation matrix of recycling value for the rock debris samples in this tunnel section, the proximity degree of recycling value for a single rock debris sample can be calculated according to Eqs. (2)–(8).

(3) Grade boundaries for the recycling value of rock debris

Taking into account the varying performance demands for the recycling of rock debris, *UCS*, *AFC*, *MC*, and *CI* are categorized into different grades. By incorporating these graded boundary values into Eqs. (2)–(8), we obtain the TOPSIS proximity classification boundaries, as outlined in Table 5.

**Table 5:** TOPSIS proximity and recycling value grading.

Grade indicators	Grade 1	Grade 2	Grade 3	Grade 4
*UCS*/MPa	> 70	50–70	40–50	< 40
*AFC*	< 0.010	0.010–0.015	0.015–0.050	> 0.050
*MC*	< 0.02	0.02–0.05	0.05–0.10	> 0.10
*CI*	< 0.05	0.05–0.10	0.10–0.20	> 0.20
TOPSIS proximity	< 0.4343	0.3136–0.4476	0.5902–0.7286	> 0.7286

According to Table 5, this paper classifies the recycling value of TBM tunnel rock debris into 4 grades, corresponding to the four-level processing network for rock debris.

## Analysis of tunneling parameters

In the previous context, a target function for the recycling value of TBM tunnel rock debris was established, and the value was graded using the TOPSIS method. However, the experimental measurement of performance indicators in Table 1 takes a considerable amount of time. To obtain the recycling value of the rock debris produced by the TBM cutterhead during excavation in real time, this research employs machine learning classification algorithms and utilizes TBM excavation parameters during construction to perceive the grade of the tunnel rock debris’s recycling value. To select the features of TBM excavation parameters suitable for the machine learning perception model of rock debris recycling value, it is necessary to analyze the importance of the parameters and the correlation between them.

### Analysis of weighting of tunneling parameters

To select features for machine learning, this paper employs a near-optimal feature selection algorithm, called ReliefF, to analyze the weights of tunneling parameter features. The Relief algorithm, proposed by Kira in 1992, is primarily used to address feature selection issues in binary classification. To address Relief’s inability to handle multi-class problems, Kononenko improved the Relief algorithm and proposed ReliefF^26^ The core of the ReliefF algorithm lies in the concept of weights, which calculates the weight of a feature based on its correlation with class labels. In this algorithm, the correlation between features and class labels is measured by the feature’s ability to distinguish nearby samples. The specific calculation process is as follows: For any feature, first, a sample *x*_*i*_ is randomly selected from the training set. Then, *h* nearest neighbor samples (closest to *x*_*i*_) are selected from samples of the same class as *x*_*i*_ (with the same class label), and *h* nearest neighbor samples are selected from samples of different classes from *x*_*i*_ (with different class labels). Finally, the weight corresponding to the feature is continuously updated based on the single iteration formula (9) for weights, and the calculation is repeated *g* times until all samples have been processed, resulting in the final weight for a feature. The single iteration formula for weights is as follows:

9 $$W^{{i + 1}} (f_{l} ) = W^{i} (f_{l} ) - \frac{{\sum\nolimits_{{j = 1}}^{h} {{\text{diff}}(f_{l} ,x_{i} ,H_{j} )} }}{{gh}} + \sum\limits_{{C \ne lable(x_{i} )}} {\frac{{P(C)}}{{1 - P[lable(x_{i} )]}}} \cdot \sum\limits_{{j = 1}}^{h} {\frac{{{\text{diff}}[f_{l} ,x_{i} ,M_{j} (C)]}}{{gh}}}$$

where *W*^*i*^( *f*_*l*_ ) represents the weight of the *l*-th feature in the *i*-th sample *x*_*i*_; *H*_*j*_( *j* = 1,2,3…*h*) denotes the *j*-th sample among the *g* nearest neighbors that belong to the same class as *x*_i_; *P*(*C*) indicates the proportion of samples belonging to class *C* in the training set; *P*[lable(*x*_*i*_)] represents the proportion of samples belonging to the same class as *x*_i_ among the total samples, where label(*x*_i_) is the label of *x*_i_; *M*_j_(*C*) (*j* = 1,2,3…*h*) stands for the *j*-th sample among the *k* nearest neighbors of *x*_*i*_ that belong to a different class (with class label *C*). The expression for the distance function diff( *f*, *x*_*i*_, *x*_*j*_ ) between samples *x*_i_ and *x*_*j*_ on an arbitrary feature *f* is as follows:

10 $${\text{diff}}({x_i},{x_j})=\frac{{\left| {{x_i}(f) - {x_j}(f)} \right|}}{{\hbox{max} (f) - \hbox{min} (f)}}$$

where *x*_*i*_( *f* ) represents the value of sample *x*_*i*_ on feature *f*; *x*_*j*_( *f* ) represents the value of sample *x*_*j*_ on feature *f*; max( *f* ) and min( *f* ) represent the maximum and minimum values of feature *f* on ***X***^m^, respectively.

The ReliefF method is adopted to calculate the weights of the TBM tunneling parameter features in the identification of rock debris recycling value grades, as shown in Table 6.

**Table 6:** Weights of the TBM tunneling parameters.

Features	T	Ph	PRev	Tr	Ar	Pc	Pb
*W*(*f*)	0.0987	0.1404	0.0599	0.1195	0.0399	0.0050	0.0911

According to Table 6, the cutterhead power and thrust during TBM construction are relatively important factors in perceiving the recycling value of tunnel rock debris from TBM. While the role of cylinder pressure is relatively minor, and it can be excluded to reduce model dimension.

### Analysis of correlation among tunneling parameters

The purpose of feature selection is not only to reduce the dimensionality of data but also to eliminate the interference of redundant features by measuring their correlation. The stronger the correlation between two features, the stronger their redundancy and substitutability. In this paper, the Pearson correlation coefficient is used to analyze the seven tunneling parameter features during the TBM construction period.

The Pearson correlation coefficient is defined as the correlation coefficient between rank variables. For a sample of size *n*, the correlation coefficient between various features is calculated as follows:

11 $${r_{xy}}=\frac{{\sum\nolimits_{{i=1}}^{n} {({x_i} - \bar {x})({y_i} - \bar {y})} }}{{\sqrt {\sum\nolimits_{{i=1}}^{n} {({x_i} - \bar {x})\sqrt {\sum\nolimits_{{i=1}}^{n} {({y_i} - \bar {y})} } } } }}$$

where *r*_*xy*_ represents the degree of linear correlation between the two features ***x*** and ***y***, with *r*_*xy*_∈[-1,1]. $\bar {x}$ and $\bar {y}$ are the average values of the feature elements in ***x*** and ***y***, respectively. If *r*_*xy*_>0, it indicates a positive correlation between the two variables, that is, the larger the value of one variable, the larger the value of the other variable; if *r*_*xy*_ <0, it indicates a negative correlation between the two variables; when *r*_*xy*_ =0, it means that ***x*** and ***y*** are uncorrelated. The larger the absolute value of the correlation coefficient, the stronger the correlation; the closer the absolute value of the correlation coefficient is to 0, the weaker the correlation. The Pearson correlation coefficients between the TBM tunneling parameter features is calculated, and the results are shown in Fig. 4.

**Fig. 5:**
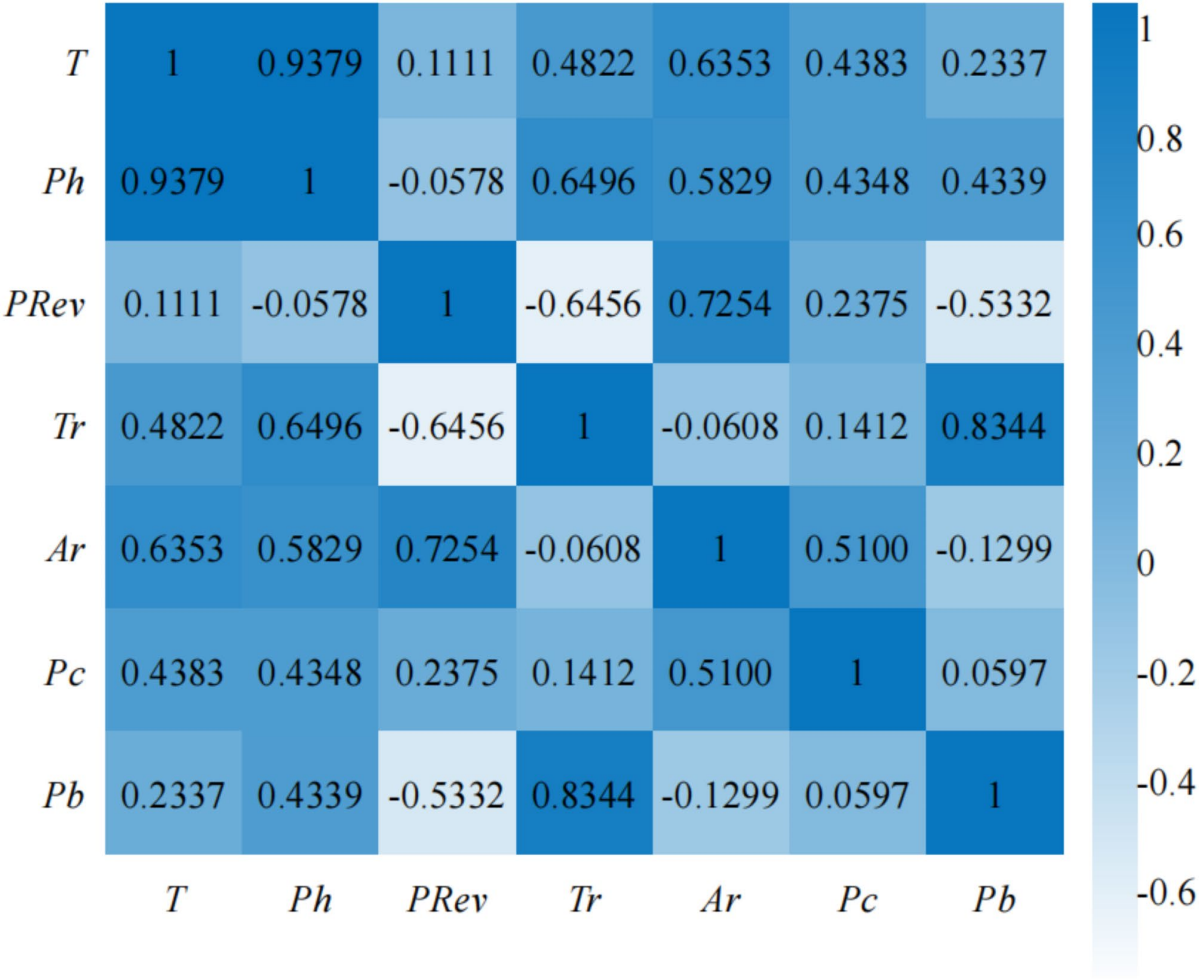
Pearson correlation coefficients between the TBM tunneling parameter features.

As shown in Fig. 5, the range of Pearson correlation coefficients among the TBM excavation parameters is [-0.6456,0.9379]. Among them, there is a strong linear correlation between the cutterhead power *Ph* and torque *T*, and one of them can be deleted to reduce the dimensionality of the model. Based on the analysis of the feature weights and correlation among the TBM tunneling parameters during the construction period, this paper selects five features, including cutterhead torque *T*, tool penetration *PRev*, cutterhead thrust *Tr*, advancing rate *Ar*, and support shoe pump pressure *Pb* to conduct real-time perception of the recycling value level of rock debris.

## Perception of rock debris recycling value levels

### Machine learning classification methods

After a thorough analysis of the characteristics of TBM excavation parameters, this research integrates four machine learning techniques, namely Classification and Regression Tree (CART), Support Vector Machine (SVM), K-nearest Neighbors (KNN), and Artificial Neural Network (ANN), to establish a real-time perception model for the recycling value levels of tunnel rock debris.

1. CART

The CART method is built on an implicit assumption that the relationship between features and the target is linear or nonlinear, and it can also be used to handle complex nonlinear relationships. In decision trees, the feature carrying the most information is automatically selected for classification, while the remaining features are rejected, thus improving computational efficiency and eliminating subjective uncertainty. The establishment of CART is based on binary recursive partitioning, which is an iterative process of dividing data into different parts. Firstly, all training samples are used to determine the structure of CART. Then, the algorithm decomposes the data using every possible binary partitioning and selects the partitioning that divides the data into two parts, minimizing the sum of squared deviations from the mean in the independent parts. Finally, the partitioning process is applied to each new branch. This process continues until each node reaches the minimum node size specified by the user.

The CART algorithm uses the Gini Index to select features, where the Gini Index represents the impurity of the model. A smaller Gini Index indicates lower impurity and a better feature. The process of classification itself is a process of reducing uncertainty, which is equivalent to enhancing purity. The purity (Gini Index) of a dataset ***D***, represented as *Gini*(***D***), is expressed as:

12 $$Gini(\mathcal{D})=\sum\limits_{{i=1}}^{n} {\left[ {p({x_i}) \cdot (1 - (p({x_i}))} \right]} =1 - \sum\limits_{{i=1}}^{n} {p{{({x_i})}^2}}$$

where *p*(*x*_i_) represents the probability of the occurrence of class *x*_i_. *Gini*(***D***) reflects the probability that two randomly selected samples from dataset ***D*** have inconsistent class labels. Therefore, the smaller the *Gini*(***D***), the higher the purity of dataset ***D***. For a sample set ***D***, it can be divided into two parts, ***D***_1_ and ***D***_2_, based on whether a certain feature *A* takes a particular value *a*. Therefore, the CART classification tree algorithm establishes a binary tree rather than a multi-way tree.

13 $${D_1}=(x,y) \in \left. D \right|A(x)=a,{D_2}=D - {D_1}$$

Under the condition of attribute *A*, the *GiniIndex* of sample ***D*** is defined as

14 $$GiniIndex(\left. \mathcal{D} \right|A=a)=\frac{{\left| {{\mathcal{D}_1}} \right|}}{\mathcal{D}}Gini({D_1})+\frac{{\left| {{\mathcal{D}_2}} \right|}}{\mathcal{D}}Gini({\mathcal{D}_2})$$

The algorithm starts from the root node and recursively builds a classification tree using the training set: (1) For the current node’s dataset ***D***, if the number of samples is less than the threshold or there are no features, a decision subtree is returned, and the current node stops recursing. (2) Calculate the Gini Index of the sample set ***D***. If the Gini Index is less than the threshold, a decision subtree is returned, and the current node stops recursing. (3) Calculate the Gini Index for each value of each existing feature at the current node. (4) Among the calculated Gini Index for each value of a feature, select the feature with the smallest Gini Index and its corresponding value as the optimal feature and optimal splitting point. Then, based on the optimal feature and optimal splitting point, divide the dataset of the current node into two parts. (5) Recursively call steps 1) to 4) for the left and right child nodes to generate the CART classification tree. When making predictions with the generated CART classification tree, if a sample from the test set falls into a leaf node and there are multiple training samples in that node, the category of the test sample is determined as the category with the highest probability within that leaf node.

(2) SVM

SVM is an intelligent learning algorithm based on the principle of structural risk minimization, which has incomparable advantages compared to the empirical risk minimization principle of traditional machine learning algorithms. SVM is a binary classification model, and its basic model is defined as a linear classifier with the largest margin in the feature space. Its learning strategy is to maximize the margin, which can ultimately be transformed into the solution of a convex quadratic programming problem, thus ensuring that the obtained extreme value solution is the global optimal solution. The learning objective of the linear classifier is to find a hyperplane in the n-dimensional data space, and the equation of this hyperplane can be expressed as:

15 $$\varvec{w}^{T} x + \varvec{b} = 0$$

where ***w*** is perpendicular to the normal vector of the hyperplane, and ***b*** is the intercept of the hyperplane.

**Fig. 6:**
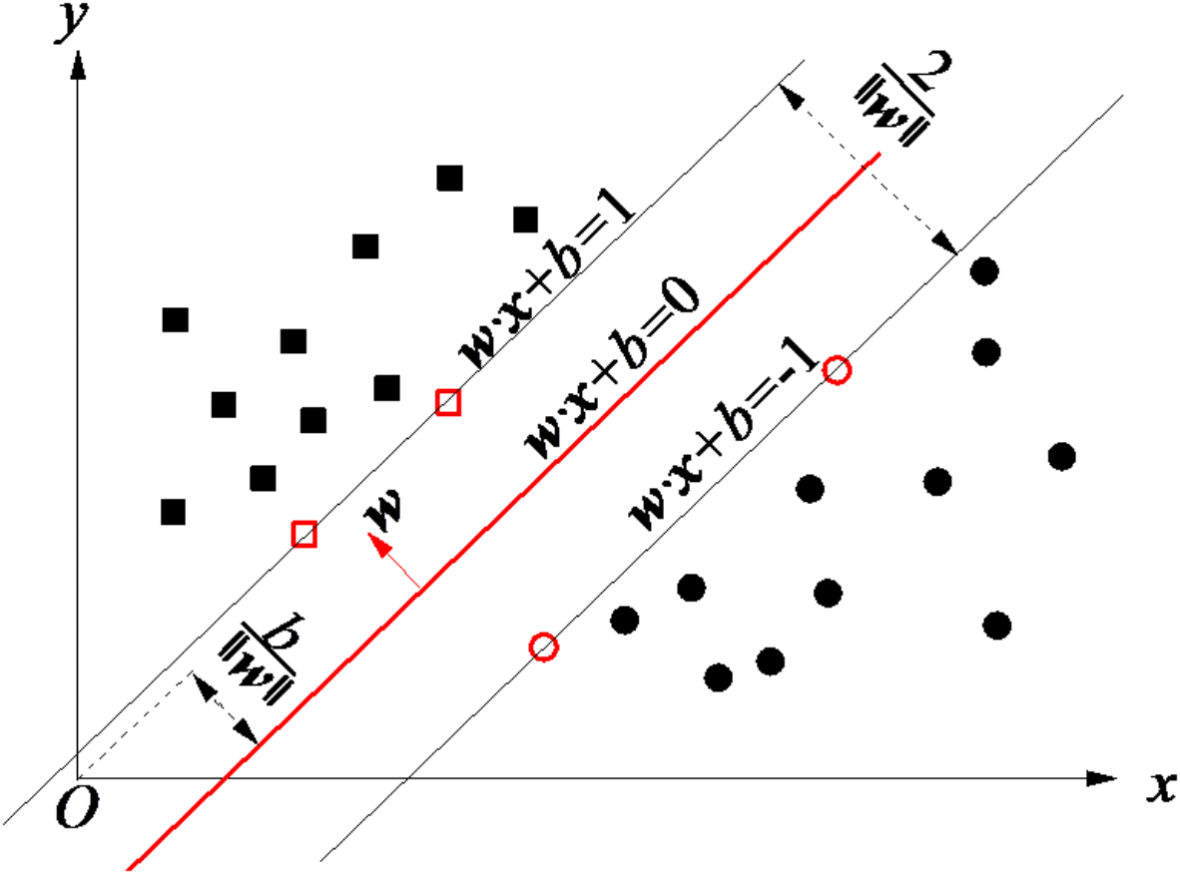
The hyperplane in the 2-dimensional data space.

As shown in Fig. 6, for the given training samples $\left\{ {({x_i},{y_i})} \right\}_{{i=1}}^{n}$, where *x*_*i*_∈***X***^m^ represents *m* tunneling parameters related to the recycling value of TBM tunnel rock debris, and *y*_*i*_ represents their recycling values. The SVM classification objective function can be expressed as:

16 $$\min \frac{1}{2}\left\| \varvec{w} \right\|^{2} \;\;s.t.,y_{i} (\varvec{w}^{T} x_{i} + \varvec{b}) \ge 1,\;\;i = 1,2, \ldots ,N$$

By using the Lagrange function, the optimization objective is transformed into an unconstrained optimization function:

17 $$L(\varvec{w},\varvec{b},\alpha ) = \frac{1}{2}\left\| \varvec{w} \right\|^{2} - \sum\limits_{{i = 1}}^{N} {\alpha _{i} } [y_{i} (\varvec{w}^{T} x_{i} + \varvec{b}) - 1]\;\;s.t.,\;\;\alpha _{i} \ge 0$$

where *α*_*i*_ represents the Lagrange multiplier, and the objective function is:

18 $$\mathop {\max }\limits_{{\alpha _{i} }} \mathop {\min }\limits_{{\varvec{w},b}} L(\varvec{w},\varvec{b},\alpha )$$

From the above formula, we can first find the minimum value of the optimization function for ***w*** and ***b***, then find the maximum value of the Lagrange multiplier *α*, and finally determine the hyperplane. Finally, the optimal hyperplane obtained is used to classify the samples mapped to the *n*-dimensional feature space, and their categories are determined based on their positions relative to the hyperplane (which side of the hyperplane they are in).

(3) KNN

The KNN (K-Nearest Neighbors) algorithm is a classification algorithm in supervised learning. For a new data point x in an n-dimensional space, the algorithm determines its category based on the categories of the K nearest neighbors. The selection of the value of K, the number of neighboring points, has a significant impact on the classification results. If *K* is set to a small value, the presence of noise components can have a significant impact on the prediction, leading to overfitting. On the other hand, if *K* is set to a large value, it is equivalent to using training instances in a larger neighborhood for prediction, which can increase the approximation error of learning. Increasing the value of *K* simplifies the overall model. A common approach is to start with *K* = 1 and estimate the error rate of the classifier using a validation set. Repeat this process, incrementing *K* by 1 each time to allow for an additional neighbor, until the *K* that produces the minimum error rate is selected. Typically, the value of *K* does not exceed 20, with an upper limit of the square root of *n*. As the dataset increases, the value of *K* should also increase. In the KNN algorithm, the distance between two points ***x*** and ***y*** can be calculated using the Euclidean distance:

19 $$d(x,y)=\sqrt {\sum\limits_{{i=1}}^{n} {{{({x_i} - {y_i})}^2}} }$$

or the Manhattan distance:

20 $$d(x,y)=\sqrt {\sum\limits_{{i=1}}^{n} {\left| {{x_i} - {y_i}} \right|} }$$

The process of the KNN algorithm is as follows: (1) Calculate the distance between each test data and training data; (2) Sort the distances in ascending order; (3) Select the *K* points with the smallest distances; (4) Determine the frequency of occurrence of the categories represented by the top *K* points; (5) Return the category with the highest frequency among the top *K* points as the predicted classification for the test data.

(4) ANN

The structure of an ANN model is highly similar to the neural structure of an animal’s brain. It receives input data, processes it through calculations, and then outputs prediction results. Typically, an ANN network model consists of an input layer, one or more hidden layers, and an output layer. Neural networks with multiple hidden layers are also known as deep neural networks. Each layer of the neural network has several neurons, and the neurons between layers are fully connected. Each neuron receives the output value of the previous layer’s neurons as its input value, processes it, and then outputs it to the neurons of the next layer. Finally, the neurons in the output layer produce the predicted result values.

**Fig. 7:**
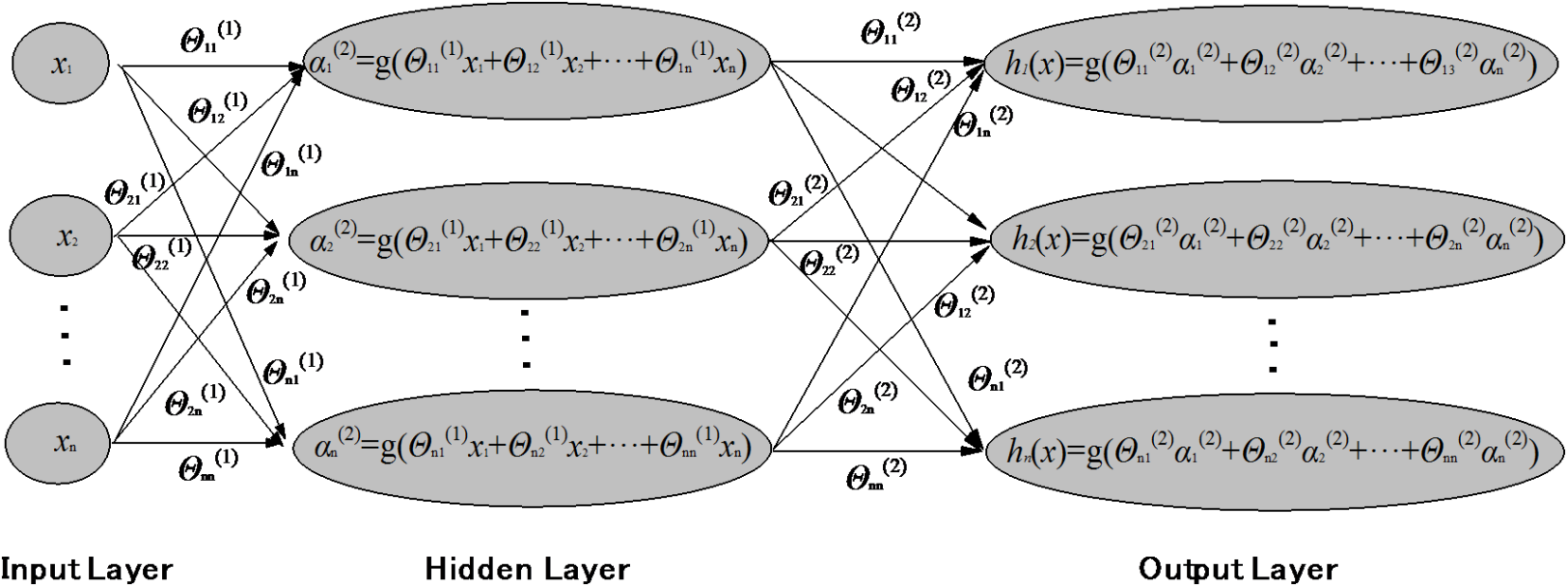
The structure of an ANN model.

As shown in Fig. 7, the neural network model consists of an input layer with *n* variables, a hidden layer, and an output layer. Here, *Θ*^j^ represents the matrix of parameters (also known as weight values) connecting neurons from layer j to layer *j* + 1, with subscripts indicating specific values within the matrix. The activation term of the *i-*th neuron in layer *j* is denoted as $\alpha _{i}^{j}$, which represents the value computed and output by that neuron. For the hidden layer and the output layer, the input values of each neuron are the collective output values of the neurons in the previous layer. These values are processed through a function to produce the output value of the neuron. The activation function of the neuron is *g*( ), which is typically the Sigmoid function.

### Optimization of hyperparameters in machine learning models

Hyperparameters in machine learning are the parameters used to control the behavior of an algorithm when building a model. These parameters cannot be learned from the normal training process and need to be assigned values before training the model. Typically, it is necessary to optimize the hyperparameters to select an optimal set of hyperparameters for the learning machine, thereby improving the performance and effectiveness of learning. Methods for hyperparameter optimization include manual tuning, grid search, random search, and Bayesian optimization. Manual tuning is time-consuming and labor-intensive, and it does not guarantee the best combination of parameters. Grid search can create models by permuting and combining all hyperparameter values, and then evaluate and select the best model. However, this requires cross-validation of parameter permutations and combinations, resulting in slow optimization speed. Since not all hyperparameters may be equally important, random search randomly selects parameter combinations from the hyperparameter space and selects them based on the given number of iterations. Bayesian optimization finds the value that minimizes the objective function by establishing a surrogate function (probability model) based on past evaluations of the objective function. The difference between the Bayesian method and random or grid search is that it takes into account previous evaluation results when trying the next set of hyperparameters, thus avoiding a lot of unnecessary work. Therefore, Bayesian hyperparameter optimization boasts significant efficiency advantages, including efficient global search capabilities, balanced exploration and exploitation, reduced computational costs for expensive objective functions, interpretability, and robustness to noise and irregularity.

**Fig. 8:**
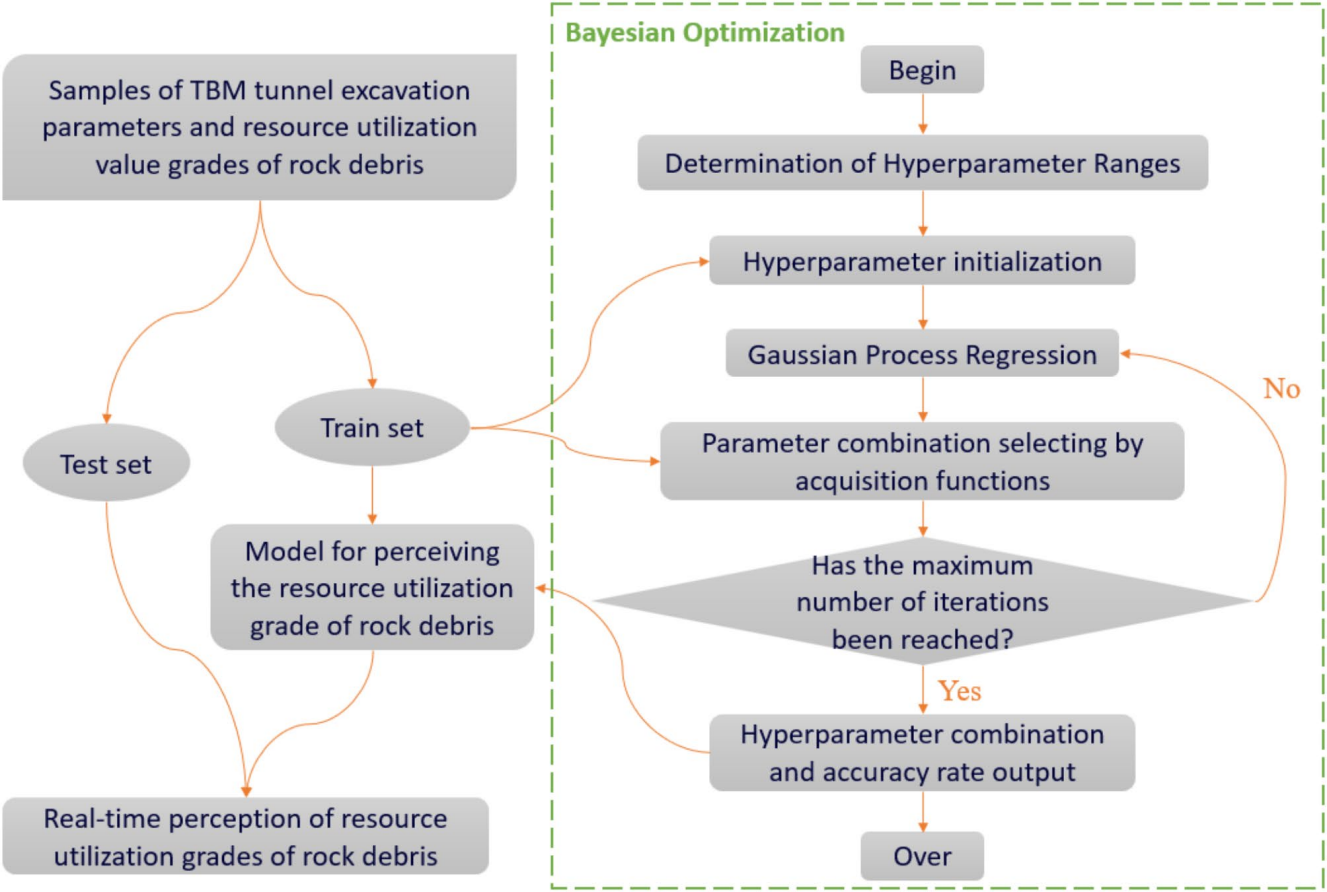
The Bayesian optimization process.

The Bayesian optimization process is illustrated in Fig. 8. Firstly, parameter combinations are randomly generated based on the types and ranges of hyperparameters. The machine learning model is then trained using a sample training set. After that, the model’s accuracy is calculated using a 5-fold cross-validation method as the optimization evaluation criterion, and the result is used to refine the model through a probabilistic surrogate model such as a Gaussian process. Subsequently, acquisition functions such as Probability Improvement (PI), Expectation Improvement (EI), and Upper Confidence Bound (UCB) are employed to select the hyperparameter combinations within the Gaussian process, and the model accuracy under those combinations is calculated. This process is repeated until the maximum number of iterations is reached. Finally, the hyperparameter combination and the corresponding model accuracy are output, and the perception model under that hyperparameter combination is adopted as the Bayesian optimization model.

In this research, the Bayesian method is employed to optimize the hyperparameters of the four types of machine learning algorithms described in 5.1. The types of hyperparameters involved, the ranges of parameter values, the optimized values, and the optimal acquisition functions are presented in Table 7.

**Table 7:** The optimized values of the hyperparameters and the optimal acquisition functions.

Algorithms	Hyperparameters	Ranges	Optimized values	Acquisition functions
CART	Maximum number of splits	1–79	14	EI
	Split criterion	Gini’s diversity index; Towing rule; Maximum deviance reduction	Gini’s diversity index	
SVM	Multiclass method	One-vs-All; One-vs-One	One-vs-One	EI
	Box constraint level	0.001–1000	86.4007	
	Kernal function	Gaussian; Linear; Quadratic; Cubic	Quadratic	
KNN	Number of neighbors	1–40	1	UCB
	Distance metric	City block; Chebyshev; Correlation; Cosine; Euclidean; Minkowski; Hamming, et al.	Chebyshev	
	Distance Weight	Equal; Inverse; Squared inverse	Squared inverse	
ANN	Number of fully connected layers	1–3	2	PI
	Activation	ReLU; Tanh; Sigmoid; None	Tanh	
	Regularization strength (Lambda)	1.25e-7–1250	9.31e-5	
	First layer size	1–300	123	
	Second layer size	1–300	139	

This research utilizes four types of machine learning algorithms (ANN, SVM, KNN, and CART) after Bayesian hyperparameters optimization to establish a perception model for the recycling value of TBM tunnel rock debris. This model enables real-time identification of the recycling value of tunnel rock debris based on TBM excavation parameters during the TBM construction period. After the perception process, the 5-fold cross-validation was conducted to evaluate the performance of the models. is a method that randomly splits the dataset into 5 equal parts, trains the model on 4 parts and tests it on the remaining 1 part, repeating this process 5 times to ensure that each subset is used as the test set once. The computational cost is moderate due to traditional algorithms and modest data processing volumes. The latency is low, typically within milliseconds to tens of milliseconds per sample, ensuring timely decision-making. The models require a modern multi-core CPU, 8–16 GB RAM, and adequate SSD storage. No specialized hardware (e.g., high-end GPUs) is necessary. They can provide predictions in sub-millisecond to millisecond ranges, which is suitable for real-time applications in TBM operations.

**Fig. 9:**
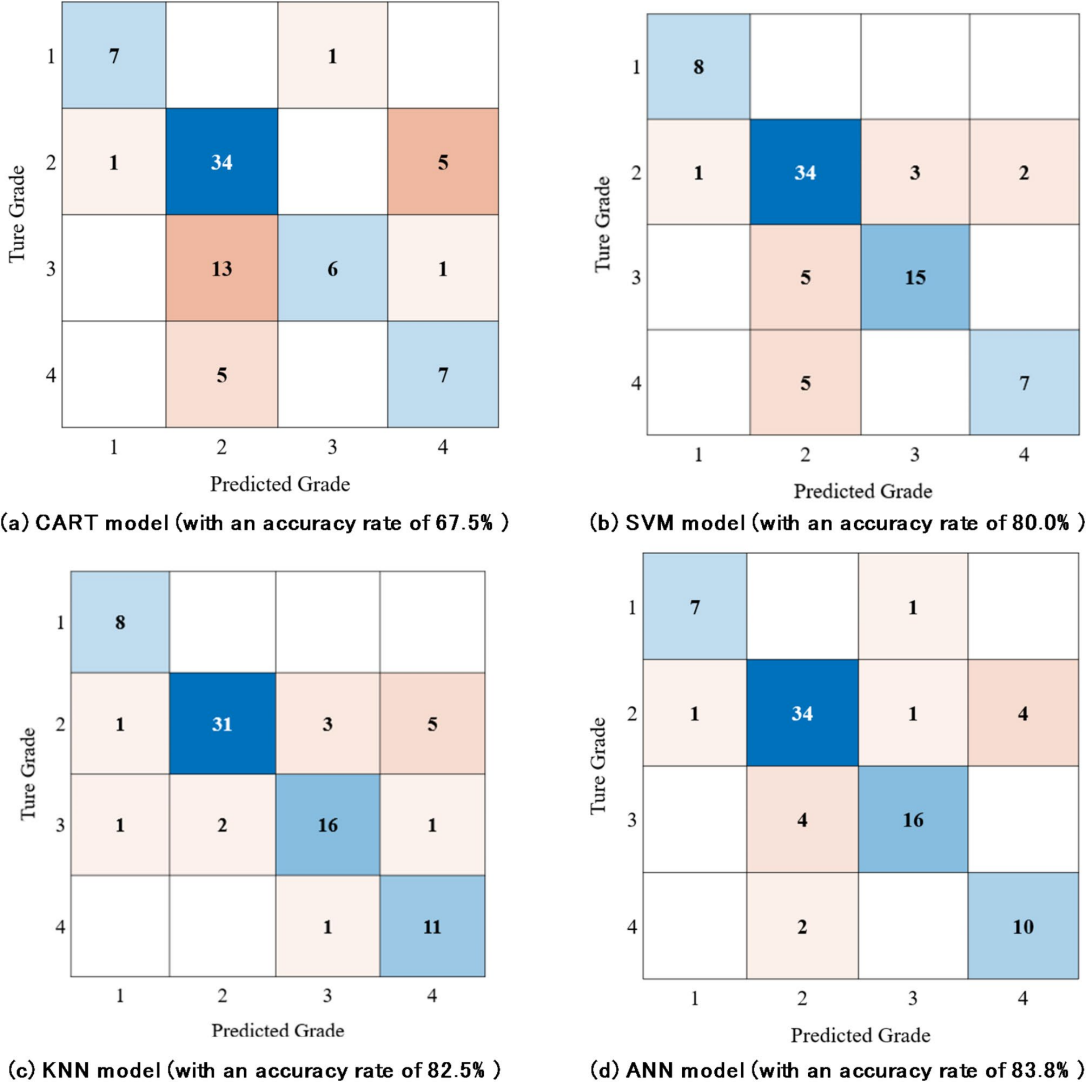
The confusion matrices and recognition accuracies of the perception models.

The confusion matrices and recognition accuracies of the perception models based on different algorithms after 5-fold cross-validation are shown in Fig. 9.

As shown in Fig. 9, the recognition accuracy rates of the perception model for recycling value grades of TBM tunnel debris, based on the hyperparameter-optimized CART, SVM, KNN, and ANN algorithms, are 67.5%, 80.0%, 82.5%, and 83.8%, respectively. Among them, there are a relatively large number of samples identified as recycling value grade 2, while fewer samples are identified as grades 1 and 4. For the CART model and KNN model, 5 samples with grade 2 were mistakenly perceived as grade 4. For the SVM model, 10 samples with grade 3 or grade 4 were mistakenly perceived as grade 2. Relatively, the result of the ANN model, although has misconceptions about samples with grade 2, is more acceptable.

**Fig. 10:**
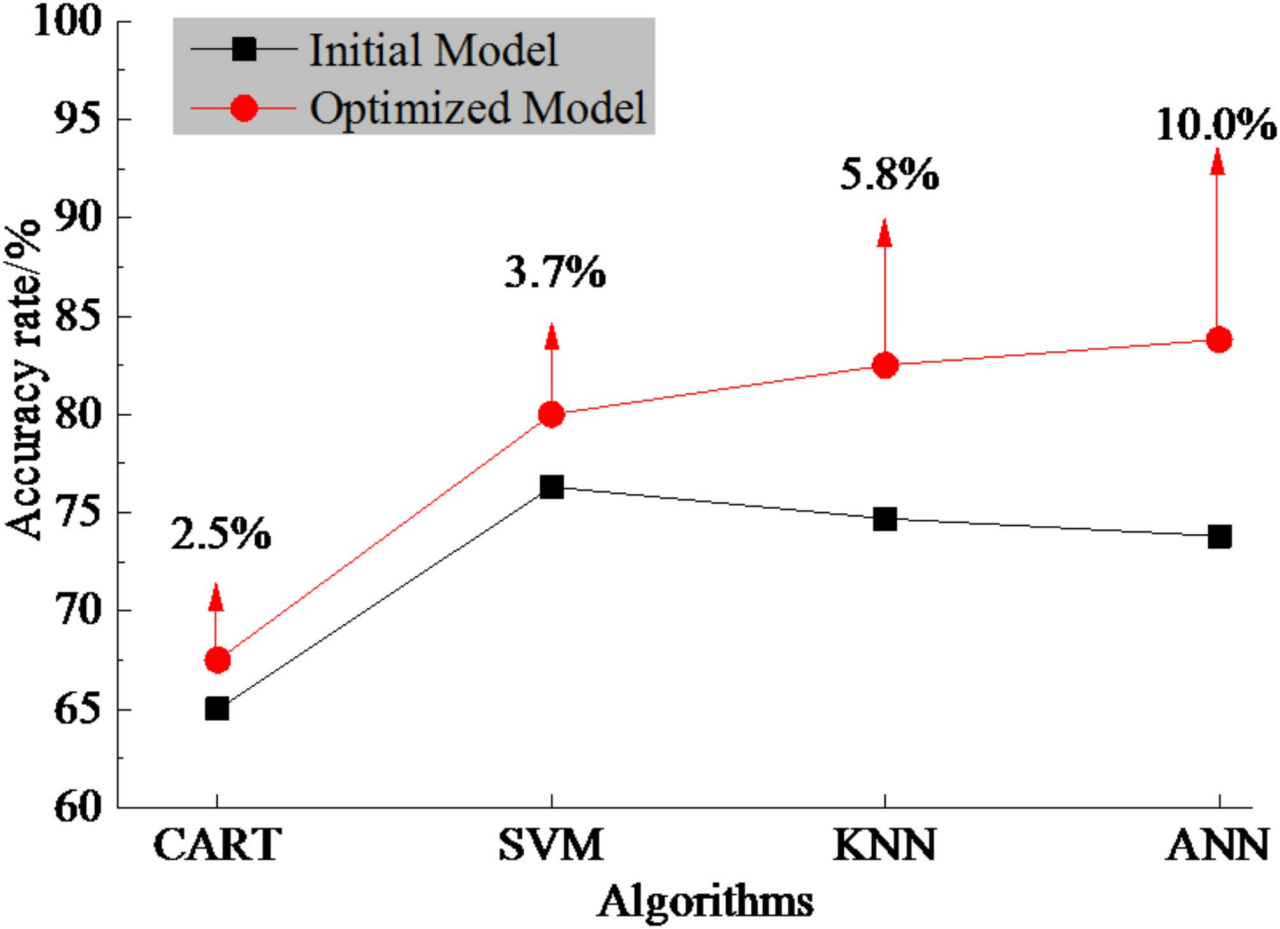
The accuracy rates before and after Bayesian hyperparameter optimization.

To validate the effectiveness of the Bayesian hyperparameter optimization process in improving the accuracy of the perception model for recycling value grades of TBM tunnel debris, the 5-fold cross-validation accuracy rates of the four types of machine learning models for rock debris recycling value grades were calculated before and after optimization, as shown in Fig. 10.

According to Fig. 10, the accuracy rates of the perception models for rock debris recycling value grades based on the CART, SVM, KNN, and ANN algorithms have improved by 2.5%, 3.7%, 5.8%, and 10% respectively after hyperparameter optimization. Notably, the Bayesian hyperparameter optimization process has a more significant effect on improving the accuracy of the perception model based on the ANN algorithm. In addition, for the ANN model, Bayesian hyperparameter optimization achieved higher accuracy (92.3% vs. 90.1%/89.7%) with significantly lower resource usage (15.8 vs. 28.5/42.0 GPU-hours) compared to random/grid search.

### Engineering application and model validation

**Fig. 11:**
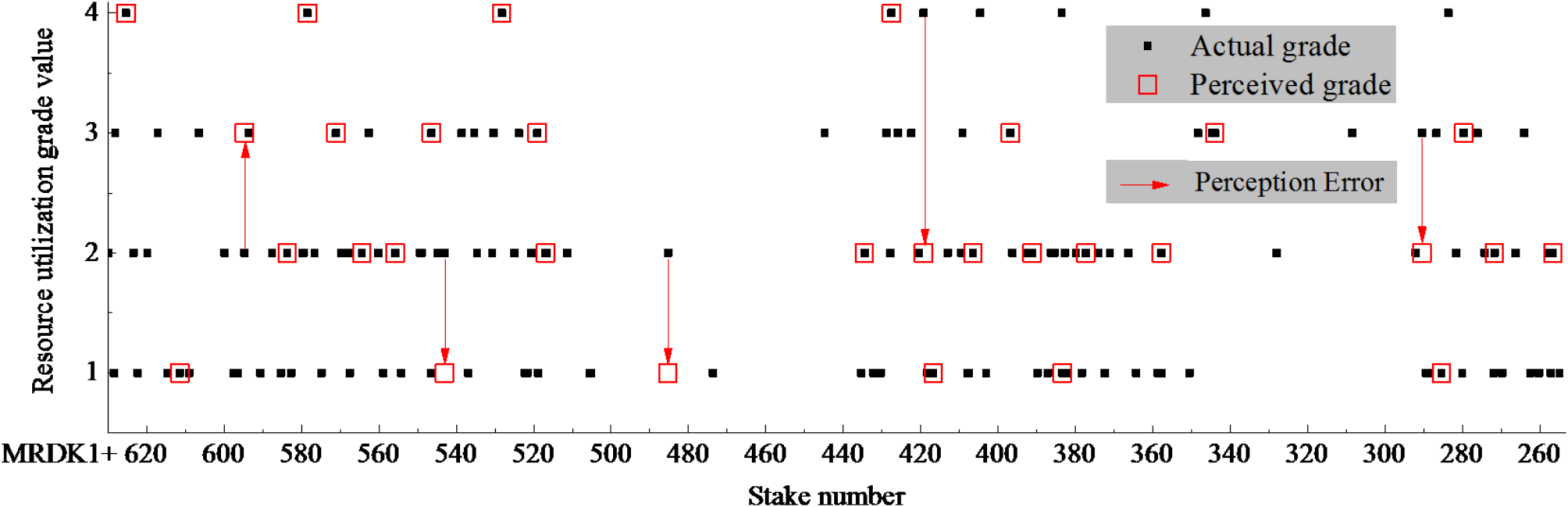
Real-time perception of the recycling value level of the rock debris during TBM construction.

Taking the train access tunnel section (spanning from stake number MRDK1 + 630 to MRDK1 + 250) of the subway Line 6 in Shenzhen as the engineering application object, this research aims to establish a machine learning model based on an optimized ANN algorithm. This model will be informed by the analysis of TBM tunneling parameters during the construction phase, as detailed in s 3.1 of this article, to enable real-time perception of the recycling value level of the rock debris in this specific tunnel section. To achieve this, we selected 150 sets of rock debris (including 30 test samples) during the TBM construction period and used our model to perceive and identify the corresponding recycling value grade of the rock debris. The perception result is graphically represented in Fig. 11.

As shown in Fig. 11, among the 30 groups of samples for the perception test, 25 sets correctly perceived the recycling value grade, with an overall perception accuracy rate of 83.3%. Therefore, in the TBM tunneling project, it is feasible to perform a continuous perception of the recycling value grade of TBM tunnel rock debris using tunneling parameters based on the optimized ANN algorithm model. Without the need for time-consuming testing, the rock debris from the project was quickly sorted and stacked, and then processed according to the treatment network.

## Discussion

Due to the complex relationship between the rock debris performance indicators and the TBM tunneling parameters, perception error occurs when the complexity of the model is insufficient to capture the fundamental relationships of the data. Furthermore, errors in performance indicator testing can also lead to perception errors. Considering the demand for real-time perception of rock debris grade and computing speed, the perception accuracy rate of the optimized ANN algorithm model in this research is acceptable.

For other TBM projects that should use this model to perceive the recycling value grade of rock debris, especially those with significant differences in geological conditions and project cases introduced in this article, it is necessary to collect test data on rock debris performance indicators and TBM tunneling parameters in the 300 to 400-meter tunnel section to train the model until the perception accuracy meets the requirements.

By pure data-driven perception and avoiding time-consuming rock physical and mechanical tests, the method proposed can guide the classified storage and disposal of tunnel rock debris, eliminating the need for tedious rock debris performance tests, avoiding the situation where rock debris of different components and properties are mixed and cannot be utilized, improving the recycling speed and rate of the tunnel rock debris, reducing the exploitation of natural stones, and achieving considerable economic and ecological benefits.

## Conclusions

This research explored the four-level processing network for tunnel rock debris during TBM tunneling and graded the recycling value of rock debris by calculating the weight and similarity of their performance indicators through the TOPSIS method. Based on a comprehensive database that encompasses both the performance indicators of tunnel rock debris and the tunneling parameters of TBM, perception models have been constructed using different machine learning algorithms, and the optimized ANN-based rock debris recycling value grade perception model was applied to a TBM tunnel project. The main conclusions of this research are drawn as follows:

1. This research considers the four-level processing network for tunnel rock debris during TBM tunneling from the three perspectives (processing cost, productivity rate, unit price and transportation cost), establishes an objective function for the recycling value of TBM tunnel rock debris, and grades the recycling value by calculating the weight and similarity of their performance indicators (uniaxial compressive strength, content of acicular and flattened particles, mud content and crushing index) through the TOPSIS method.
2. The correlation and weights of the TBM tunneling parameters during TBM construction are analyzed, and five characteristics including cutterhead torque, tool penetration, cutterhead thrust, advancing rate, and support shoe pump pressure are selected to conduct real-time perception of the recycling value level of rock debris.
3. Leveraging a comprehensive database that encompasses both the performance indicators of tunnel rock debris and the tunneling parameters of TBM, perception models are constructed using different machine learning algorithms.
4. After Bayesian hyperparameter optimization, the 5-fold cross-validation accuracy of perception models based on CART, SVM, KNN, and ANN is 67.5%, 80.0%, 82.5%, and 83.8% respectively. Among them, the hyperparameter optimization significantly improved the accuracy of the ANN perception model.
5. When applying the optimized ANN-based perception model to TBM tunnel engineering, the tested perception accuracy rate was 83.3%. This model can assist in the classified storage and processing of tunnel rock debris and improve the recycling speed and rate of tunnel rock debris.
6. For TBM projects with significant differences in geological conditions and project cases introduced in this article, test data on rock debris performance indicators and TBM tunneling parameters in the 300 to 400-meter tunnel section must be collected to train the model until the perception accuracy meets the requirements.

## Data Availability

The datasets used and analyzed during the current study are available from the corresponding author on reasonable request.

## References

[1] Yagiz, S. & Karahan, H. Application of various optimization techniques and comparison of their performances for predicting TBM penetration rate in rock mass. *Int. J. Rock. Mech. Min.* **80**, 308–315 (2015).

[2] Min, F. et al. Experimental study on property change of slurry and filter cake of slurry shield under seawater intrusion. *Tunn. Undergr. Sp. Tech.* **88**, 290–299 (2019).

[3] Su, W. et al. Analysis of the disc cutter cutting load and performance during TBM tunneling in soft-hard varied strata through laboratory cutting test. *Geotech. Test. J.* **46**, 712–730 (2023).

[4] Zhang, W. et al. Review of tunnels and tunnelling under unfavourable geological conditions. *Geol. J.* **59**, 2668–2689 (2024).

[5] Kilic, K. et al. Soft ground micro TBM Jack speed and torque prediction using machine learning models through operator data and micro TBM-log data synchronization. *Sci. Rep.* **14**, 9728. 10.1038/s41598-024-60681-8 (2024). 10.1038/s41598-024-60681-8 PMC11055924 38678078

[6] Shen, S. & Xu, Y. Numerical evaluation of land subsidence induced by groundwater pumping in Shanghai. *Can. Geotech. J.* **48**, 1378–1392 (2011).

[7] Jin, D. et al. An in-tunnel grouting protection method for excavating twin tunnels beneath an existing tunnel. *Tunn. Undergr. Sp. Tech.* **71**, 27–35 (2018).

[8] Liao, S., Peng, F. & Shen, S. Analysis of shearing effect on tunnel induced by load transfer along longitudinal direction. *Tunn. Undergr. Sp. Tech.* **23**, 421–430 (2008).

[9] Fang, Y. et al. The performance of TBM disc cutter in soft strata: A numerical simulation using the three-dimensional RBD-DEM coupled method. *Eng. Fail. Anal.* **119**, 104996 (2021).

[10] Deng, L. et al. Experimental and numerical investigations on rock breaking of TBM disc cutter based on a novel platform with rotational cutting. *Rock. Mech. Rock. Eng.* **56**, 1415–1436 (2022).

[11] Sun, W. et al. Investigation on overburden thickness considering face and anti-floating stability of shallow shield tunnel. *Comput. Geotech.* **160**, 105562 (2023).

[12] Voit, K. et al. Tunnel spoil recycling for concrete production at the Brenner base tunnel in Austria. *Struct. Concr.* **21**, 2795–2809 (2020).

[13] Lin, G. et al. Application potential of granite cutting waste and tunnel excavation rock as fine aggregates in cement-based materials based on surface characteristics. *J. Build. Eng.* **62**, 105380 (2022).

[14] Luo, Q., Liu, P. & Wu, M. Re-using excavated rock from underground tunneling activities to develop eco-friendly ultrahigh performance concrete. *Case Stud. Constr. Mater.* **20**, e02867. 10.1016/j.cscm.2024.e02867 (2024).

[15] Maximilian, H. et al. Applicability of excavated rock material: A European technical review implying opportunities for future tunneling projects. *J. Clean. Prod.* **315**, 128049 (2021).

[16] Amin, S. et al. A novel extreme learning machine based kNN classification method for dealing with big data. *Expert Syst. Appl.* **183**, 115293 (2021).

[17] Seyed, M. M. et al. Predicting the fracture mechanics responses of recycled asphalt mixes using machine learning-based algorithms: Application of CART algorithm and neural networks. *Eng. Fract. Mech.* **276**, 108791 (2022).

[18] Daniel, D. et al. Comparative of machine learning classification strategies for electron energy loss spectroscopy: Support vector machines and artificial neural networks. *Ultramicroscopy* **253**, 113828 (2023). 37556961 10.1016/j.ultramic.2023.113828

[19] Hu, H., Qi, L. & Chao, X. Physics-informed neural networks (PINN) for computational solid mechanics: Numerical frameworks and applications. *Thin Walled Struct.* **205**, 112495. 10.1016/j.tws.2024.112495 (2024).

[20] Li, Q. et al. Intelligent identification and warning method of disc cutter abnormal wear in TBM construction based on extreme learning machine. *Sci. Rep.* **14**, 30655. 10.1038/s41598-024-76172-9 (2024). 39730385 10.1038/s41598-024-76172-9 PMC11680588

[21] Liu, J. et al. Machine learning-based techniques for land subsidence simulation in an urban area. *J. Environ. Manag.* **352**, 120078. 10.1016/j.jenvman.2024.120078 (2024). 10.1016/j.jenvman.2024.120078 38232594

[22] Xue, Y. et al. An intelligent method for TBM surrounding rock classification based on time series segmentation of rock-machine interaction data. *Tunn. Undergr. Sp. Tech.* **140**, 105317 (2023).

[23] Shen, X. et al. Prediction of the slurry pressure and inversion of formation characteristics based on a machine learning algorithm during tunnelling in a fault fracture zone. *Tunn. Undergr. Sp. Tech.* **144**, 105514. 10.1016/j.tust.2023.105514 (2024).

[24] Li, C., Zhou, J. & Du, K. Towards lightweight excavation: Machine learning exploration of rock size distribution prediction after tunnel blasting. *J. Comput. Sci.* **78**, 102266. 10.1016/j.jocs.2024.102266 (2024).

[25] Ministry of Railways. *Standard for Constructional Quality Acceptance of Railway Concrete Engineering (TB 10424-2010) * 26–28 (China Railway Publishing House, 2011).

[26] Robink-Šikonja, M. & Kononenko, I. Theoretical and empirical analysis of ReliefF and RReliefF. *Mach. Learn.* **53**, 23–69 (2003).

